# Chronic intermittent hypoxia disrupts protective microgliosis in ischemic proliferative retinopathy

**DOI:** 10.1186/s12974-025-03392-9

**Published:** 2025-03-14

**Authors:** Tianxiang Yang, Kaitryn E. Ronning, Sébastien Augustin, Frédéric Blond, Caroline Nous, Foteini Argyriou, Sara Touhami, Cécile Delarasse, Xavier Guillonneau, Florian Sennlaub

**Affiliations:** 1 Sorbonne University, INSERM, CNRS, Institut de la Vision, 17 rue Moreau, Paris, F-75012 France; 2 Aier Eye Institute, Changsha, Hunan Province 410015 China; 3 Centre Hospitalier National d’Ophtalmologie des Quinze-Vingts, INSERM-DHOS CIC 503, Paris, France; 4 Pitié Salpêtrière University Hospital, Sorbonne Université, 47-83 Boulevard de l’Hôpital, Paris, 75013 France

**Keywords:** Hypoxia, Ischemic retinopathy, Microglia, Sleep apnea

## Abstract

Sleep apnea that leads to chronic intermittent hypoxia (CIH) is an independent risk factor for advanced, debilitating ischemic proliferative retinopathies, such as diabetic retinopathy (DR) and retinopathy of prematurity (ROP). The underlying mechanisms are unknown. Here we investigated the consequences of CIH on the ischemic retina of the oxygen-induced retinopathy model. We show that experimental CIH inhibited colony stimulating factor 1 (CSF1) expression, blunting the reactive microgliosis during the ischemic phase of OIR. CIH severely delayed beneficial revascularization of the ischemic retina and increased pathological neovascularization. CIH also induced photoreceptor segment thinning and accentuated OIR-induced inner and outer retinal functional deficits. Mechanistically we demonstrated that local CSF1R inhibition during ischemic retinopathy reduced the number of microglial cells, inhibited revascularization, and exacerbated pathological neovascularization, recapitulating the effects of CIH. Our findings provide a novel mechanism by which sleep apnea and CIH aggravate ischemic retinopathies, underscoring the importance of treating apnea in DR and ROP to help prevent sight threatening severe disease.

**Supplementary Information:**

The online version contains supplementary material available at 10.1186/s12974-025-03392-9.

## Background

Ischemic proliferative retinopathies, such as retinopathy of prematurity (ROP) and diabetic retinopathy (DR), remain a major threat to vision despite major advances in the treatment of late neovascular complications by anti-VEGF therapies [1–3]. They are characterized by an ischemic first stage, in which there is retinal microvascular degeneration and associated neuronal degeneration. This occurs in ROP due to the premature interruption of the maternal metabolic-endocrine support of the immature fetus with exposure to supra-physiological extra-uterine oxygen levels, and in diabetes due to metabolic dysregulation. In both diseases this first stage can evolve into advanced stages characterized by neovascularization that can lead to inner retinal edema and fibrosis [1–3]. In addition to neovascularization, pathological neuronal degeneration occurs in the inner retina [4–6], photoreceptor segments are shortened and their structure is perturbed in DR [6–9], and photoreceptor dysfunction remains detectable years after the preterm days of ROP patients [10].

Interestingly, the risk of severe ROP is increased in infants suffering from apnea of prematurity [11, 12], and decreased when the apnea is treated with caffeine [13]. Similarly, obstructive sleep apnea, characterized by recurrent upper airway collapse and interruptions in breathing during sleep, is independently associated with an increased prevalence of advanced DR, proliferative DR, and diabetic macular edema (DME) after adjustment to confounders including age, sex, body mass index, and hypertension [14–16]. Of note, sleep apnea is also associated with cardiovascular disease [17] and in particular the risk of stroke [18]. Sleep apnea produces repeated episodes of hypoxemia followed by re-oxygenation, called chronic intermittent hypoxia (CIH), which is believed to be the major contributor to sleep apnea-related morbidity and mortality [19].

Ischemic proliferative retinopathy can be experimentally induced in mice by exposing pups to 75% O_2_ for five days during their second week of life. This oxygen-induced retinopathy (OIR) model [20] provokes vaso-obliteration and subsequent capillary loss of the central retinal vasculature leading to an ischemic retina when the mice are returned to room-air conditions. The oxygen-induced central avascular area subsequently, and invariably, triggers VEGF-dependent growth of pathological intravitreal neovascular tufts [20] that reach a maximum a further five days into the ischemic phase of the model (PN 17–18). In parallel, the avascular area disappears at the ischemic retina revascularizes and the neovascular tufts resorb by PN25 [21]. This phenomenon is also observed in most ROP patients. Intra-retinal reperfusion and revascularization of the ischemic retina has also been observed in DR [22–24], but is generally outweighed by continued microvascular degeneration. In rodents, this revascularization has been shown to be at least in part dependent on microglial cells, the resident macrophages of the central nervous system [25, 26].

Contrary to the mild retinal changes of animal models of diabetes [27], the mouse OIR model results in sizeable ischemic avascular zones and neovascularization as in severe human DR and ROP. The clear separation of the hyperoxic, vaso-obliterative phase from the ischemic phase in the mouse OIR model also helps to specifically address mechanisms in the ischemic retina compared to rat models in which oscillating hyper- and hypoxic exposures are necessary to trigger pathological changes [28].

To investigate how CIH influences ischemic proliferative retinopathy we exposed mice with oxygen-induced ischemic retinopathy to experimental CIH. The CIH was simulated by exposing experimental animals to 10 h daily of 266 cycles of quick oscillations of the O_2_ concentration in the cage during their sleep, mimicking CIH in severe obstructive sleep apnea [29]. Here we show that experimental CIH greatly delayed beneficial revascularization of the ischemic retina, and increased pathological neovascularization. CIH also induced shortening of photoreceptor segments and affected neuronal signaling with lasting functional consequences. Additionally, CIH blunted the increase of microglial cells otherwise observed in the ischemic retina. We demonstrate that the CIH-associated reduced microglial cell count is coincident with diminished colony stimulation factor 1 (CSF-1) expression, known to control microglial cell populations. Local inhibition of CSF1R, the CSF-1 receptor, during ischemic retinopathy reduced the number of microglial cells and inhibited revascularization similar to CIH. Together, these findings provide a mechanistic explanation of how apnea and CIH aggravate ischemic proliferative retinopathies and emphasize the importance of monitoring and treating apnea in DR and ROP to help prevent severe, sight-threatening disease.

## Methods

### Animals

C57BL/6J pups and nursing mothers were obtained from Charles River. All mice were housed in pathogen-free conditions with water and chow available ad libitum. All experimental protocols and procedures were approved by the French Ministry of Higher Education, Research and Innovation (APAFIS authorization #43233 2023042416144897). Cages were randomly assigned to experimental groups.

### Oxygen-induced retinopathy and chronic intermittent hypoxia

In an adaptation of the widely used OIR model [20] C57BL/6J mice at postnatal (PN) day 8 (PN8) were exposed, with their mothers, for 5 days to hyperoxic conditions (75% O_2_), inducing vaso-obliteration and subsequent capillary loss of the central retinal vasculature [20]. At PN13, mice were returned to room-air conditions which induces extensive neovascularization in all experimental mice with maximum effects on PN18.

CIH was induced in C57BL/6J mice using a Hycon0520 simulator [30] that can oscillate the cage O_2_ concentration extremely quickly. For 10 h during the daytime (corresponding to the mouse sleep period) CIH experimental animals were exposed daily to 266 cycles of a 135s program of oscillating O_2_ concentration as follows: decrease of 20.95% O_2_ to 5% O_2_ in 45s, 5% O_2_ for 30s, increase to 20.95% O_2_ in 15s, 45s of 20.95% O_2_ (adapted from Xu et al. [29]). Control animals were exposed to the same flow and noise of gasses, without affecting O_2_ concentration.

### Immunohistochemistry

Eyes were immediately placed in 4% PFA (ThermoFisher) following CO_2_ narcosis and cervical dislocation, and eyes were fixed at room temperature for 45 min. After fixation, eyes were transferred to PBS (Gibco), the anterior segments and connective tissues were removed, four curvature-relieving cuts were made into the eye cups, and the retinas were gently separated from the RPE/choroid/sclera. Retinas and RPE/choroid/sclera complexes were first incubated overnight at room temperature with AlexaFluor488-conjugated isolectin (diluted 1:200, Invitrogen) and goat or rabbit anti-IBA1 (diluted 1:400, final concentration 1.25 ug/ml, Abcam goat antibody, Wako rabbit antibody) in PBS with 0.1% Triton. Tissues were then rinsed with PBS and then incubated for 2 h at room temperature with a AlexaFluor647-conjugated donkey anti-goat or anti-rabbit IgG antibody (diluted 1:400, final concentration 5 ug/ml, Invitrogen). After final rinses in PBS, retinas and RPE/choroid/sclera complexes were mounted on glass slides with Fluoromount aqueous mounting media (Sigma). Images were captured with a Leica DM550B fluorescence microscope (Leica Biosystems). Image analysis was performed using FIJI [31].

### Flow cytometry

For flow cytometry, retinas were collected in PBS (Gibco) and placed on ice immediately following CO_2_ narcosis and cervical dislocation. Retinas were first enzymatically dissociated by incubation with Liberase TL (final concentration 0.15 mg/ml, Roche) at 37 C for 30 min, and then dissociation was completed mechanically by gently pipetting with a P1000 pipette. The resulting cell suspensions were filtered by passing through a 70 μm filter (Miltenyi) and rinsed with PBS. Cells were labelled with a viability dye (Viobility 405/520 fixable dye, diluted 1:100, Miltenyi) following manufacturer recommendations for 15 min at room temperature. After a PBS wash, cells were then labelled for 30 min on ice with the relevant flow cytometry antibodies: VioBlue-conjugated anti-mouse CD45, APC-conjugated anti-mouse CD11b, FITC-conjugated anti-mouse MHCII, PE-conjugated anti-mouse CD31, and PE-Vio770-conjugated anti-CD115 (all antibodies diluted 1:50 in PBS for a final concentration of 3 µg/ml, all purchased from Miltenyi). Finally, cells were fixed in 1% PFA (ThermoFisher). All samples were run on a FACSCelesta Cell Analyzer (BD Biosciences, USA). The software FlowJo (BD, Oregon, USA) was used for all flow cytometry analysis.

### Optical coherence tomography

Optical coherence tomography (OCT) was performed as previously described [32]. In brief, pupils were dilated using tropicamide (0.5%, Mydriaticum, Théa, France) and phenylephrine (5%, Neosynephrine, Europhta, France), and animals were anesthetized using isoflurane (Piramal, 5% to induce and under 2% to maintain anesthesia). SD-OCT scans were acquired using a Bioptigen SDIOS-HHP system fitted with an objective to allow imaging the mouse posterior segment, using the Bioptigen InVivoVue Clinic software (Bioptigen, North Carolina, USA). After acquisition, OCT volumes were exported for processing and analysis using FIJI [31]. B scans were registered to remove breathing artifacts using the MultiStackReg plugin, a modification of the TurboReg plugin [33] written by Brad Busse. Three consecutive B scans were averaged, and the retinal layers were measured manually using the measurement tools in FIJI.

### Electroretinography

After overnight dark adaptation, pupils were dilated using tropicamide (0.5%, Mydriaticum, Théa) and phenylephrine (5%, Neosynephrine, Europhta), corneas were anesthetized with oxybuprocaine eye drops (Théa, France), and animals were anesthetized with an IP injection of a mixture of ketamine (80 mg/kg, Axience) and xylazine (8 mg/kg, Bayer HealthCare). Electroretinograms (ERGs) were recorded using a Lab Cradle system fitted with a Color Dome Ganzfeld white (6500k) LED stimulator (Diagnosys). Gold wire loop corneal electrodes were placed on both eyes, a reference electrode was placed subcutaneously between the eyes, and a ground electrode was placed subcutaneously at the base of the tail. Transparent ophthalmic gel (Lubrithal, Dechra) was placed on both eyes to maintain corneal moisture and good contact with the corneal electrode throughout recordings. Animals were kept on a heating pad to maintain normal body temperature throughout experimentation. Scotopic retinal function was probed using 4 ms flashes of 3 cd*s/m^2^ with 60 s between flashes. Resulting responses were amplified and passed through a 0–300 Hz bandpass filter with a one-channel DC-/AC-amplifier. The collected waveforms were further processed using the Espion software V6 (Diagnosys) including the identification of the a- and b-wave amplitudes. A-wave amplitudes were measured from baseline to the peak of the first negative deflection, and b-waves were measured from the a-wave to the peak of the following positive deflection. A- and b-wave amplitudes were then normalized to the average normoxia a- and b-waves for presentation.

### RNA sequencing

Fresh eyes (3 eyes from the OIR-Norm group, 3 eyes from the OIR-CIH group, all at PN18) were dissected on ice to isolate retinas immediately after enucleation. Retinal RNA was extracted using the NuceloSpin RNA kit (Macherey-Nagel) according to the Midi protocol in the manufacturer’s instructions. RNA quality was confirmed using a Bioanalyzer RNA Analysis kit (Agilent). RNA sequencing libraries were prepared from 250 ng total RNA using the TruSeq Stranded mRNA Library Prep kit (Illumina) and sequenced at the Institut du Cerveau iGenSeq sequencing core facility. The RNAseq dataset generated during the current study is available in the GEO repository, accession number GSE268420.

After sequencing, fastq files were aligned to the *mus musculus* reference genome from Ensembl (Ensembl 105, December 2021) using STAR (version 2.7.9a) with the option “- -quantMode GeneCounts” to extract the raw counts for each gene, and all count files were concatenated into a single file. The count file and a descriptive sample file were loaded into our in-house R Shiny application “EyeDVseq” for analysis: the genes with a total count below 10 across samples were filtered out and the differential gene expression analysis was performed using the version 1.40.2 DESeq integrated into EyeDVseq, comparing OIR-Norm as control and OIR-CIH as the condition. The resulting gene list was then filtered, with|Log_2_(Fold Change)| > 0.5 and pAdj-values < 0.05 being considered significant. This gene list was used to perform a gene enrichment analysis using the Metascape tool [34]. Gene ontology (GO) terms were selected from the Metascape output, sorted by logP value, and filtered to remove terms with 5 or fewer associated genes in the input list.

### qPCR

Retinal RNA from 32 fresh eyes (16 eyes from the OIR-Norm group, 16 eyes from the OIR-CIH group) was collected at PN18, as above for RNA sequencing. Extracted RNA was reverse-transcribed into cDNA with the QuantiTech reverse transcription kit (Qiagen) following the quick-start protocol in the manufacturer’s instructions. The qPCR was conducted using Power SYBR Green PCR Master Mix (Applied Biosystems) on a QuantStudio 5 Real-Time PCR System (Applied Biosystems). The cycling conditions were as follows: initial denaturation at 95°C for 10 minutes, followed by 40 cycles of 95°C for 15 seconds and 60°C for 1 minute. The qPCR data was analysed using the relative quantification method (2^−ΔΔCt^) on Design & Analysis Software v2.6.0 (Applied Biosystems). The forward primer of *Csf1* was 5’-TGACCCAGGATGAGGACAGA-3’, the reverse primer of *Csf1* was 5’-GGTAGTGGTGGATGTTCCCA-3’. Both primers were custom designed by Primer-BLAST webtool, and synthesized by IDT (Integrated DNA Technology, Europe).

### Intraocular injections

Mice were anesthetized using isoflurane (Piramal, 5% to induce and under 2% to maintain anaesthesia). We injected rat anti-CSF1R (Bio-techne) or rat IgG isotype (InvivoGen) intravitreally using glass capillaries (Eppendorf) and a microinjector. Each mouse’s left eye was injected with 3 µL rat IgG isotype (10 µg/ml) as a control, and the right eye was injected with 3 µL anti-CSF1R antibody (10 µg/ml).

### Statistical analysis

All statistical comparisons were performed in Prism v9.02 (GraphPad, Massachusetts, USA). Outliers were identified using the ROUT (Robust regression and Outlier removal) method with the false discovery rate (Q) set at 1% to balance between sensitivity and specificity in outlier detection. Two group comparisons were done using a Mann-Whitney test, unpaired when comparing between animals and paired when comparing data from contralateral eyes. Multiple comparisons were performed using one- or two-way ANOVAs, as appropriate and indicated in figure legends, followed by Šídák’s multiple comparisons post hoc test. P values less than 0.05 were considered significant.

## Results

### Chronic intermittent hypoxia alone does not affect retinal vascularization or microglia cell counts

**Fig. 1:**
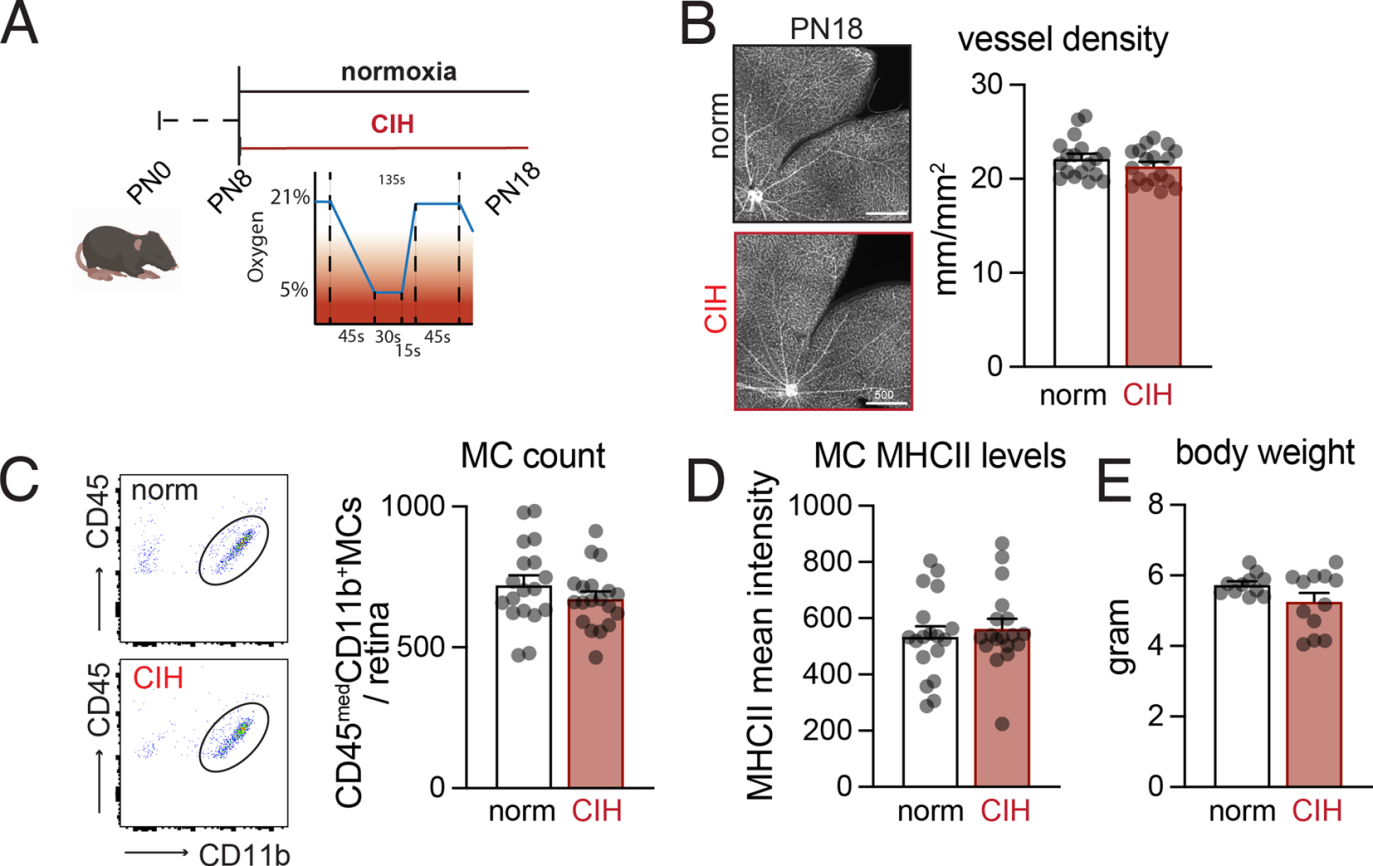
Chronic intermittent hypoxia alone does not affect retinal vascularization or microglia cell counts. (**A**) Schematic of the time course of normoxia and CIH conditions. (**B**) Left: Representative retinal flatmounts labeled with isolectin to visualize vessels. Right: Quantification of vessel density (mm vessel per mm^2^ retinal area, *n* = 18 retinas per group). (**C**) Left: Representative gating of CD45^med^CD11b^+^ microglial cells (MC). Right: Quantification of the number of microglia (single, alive, CD45^med^CD11b^+^ cells) per retina (*n* = 18–19 retinas per group). (**D**) Mean intensity of MHCII labeling on MC detected via flow cytometry (*n* = 18–19 retinas per group). (E) Body weight of pups at PN18 (*n* = 11–12 animals per group). Scale bars = 500 μm; all comparisons Mann-Whitney test, all p-values > 0.05; all values displayed as mean ± SEM, individual points indicate values per retina

To examine whether chronic intermittent hypoxia (CIH) affects retinal vasculature or microglia in the absence of retinal ischemia, pups were placed in specialized cages and exposed to CIH or normal oxygen conditions (normoxia) from postnatal (PN) day 8 to PN18. CIH was simulated by rapidly oscillating the cage O_2_ concentration for 10 h during the normal sleep hours of the animals mimicking CIH of severe obstructive sleep apnea (266 cycles over 10 h, each 135s cycle: decrease of 20.95–5% O_2_ in 45s, 5% O_2_ for 30s, increase to 20.95% O_2_ in 15s, and 45s of 20.95% O_2_; adapted from Xu et al. [29]) (schematized in Fig. 1A). Normoxic animals were exposed to the same flow and noise as CIH mice but without oscillating O_2_ concentrations. At PN18, our analysis revealed no CIH-induced alterations in vascular density in isolectin-stained retinal flatmounts (Fig. 1B), or CD45^med^CD11b^+^ cells microglia numbers in flow cytometry (Fig. 1C). Additionally, there was no difference in MHCII expression, an indicator of microglial activation, between groups (Fig. 1D). Finally, there was only a mild, non-significant reduction in body weight between groups, suggesting CIH alone does not cause growth retardation (Fig. 1E). These results demonstrate that our CIH regimen had no effect on the normal vascular maturation and development that takes place in mouse pups at this stage of development.

**Fig. 2:**
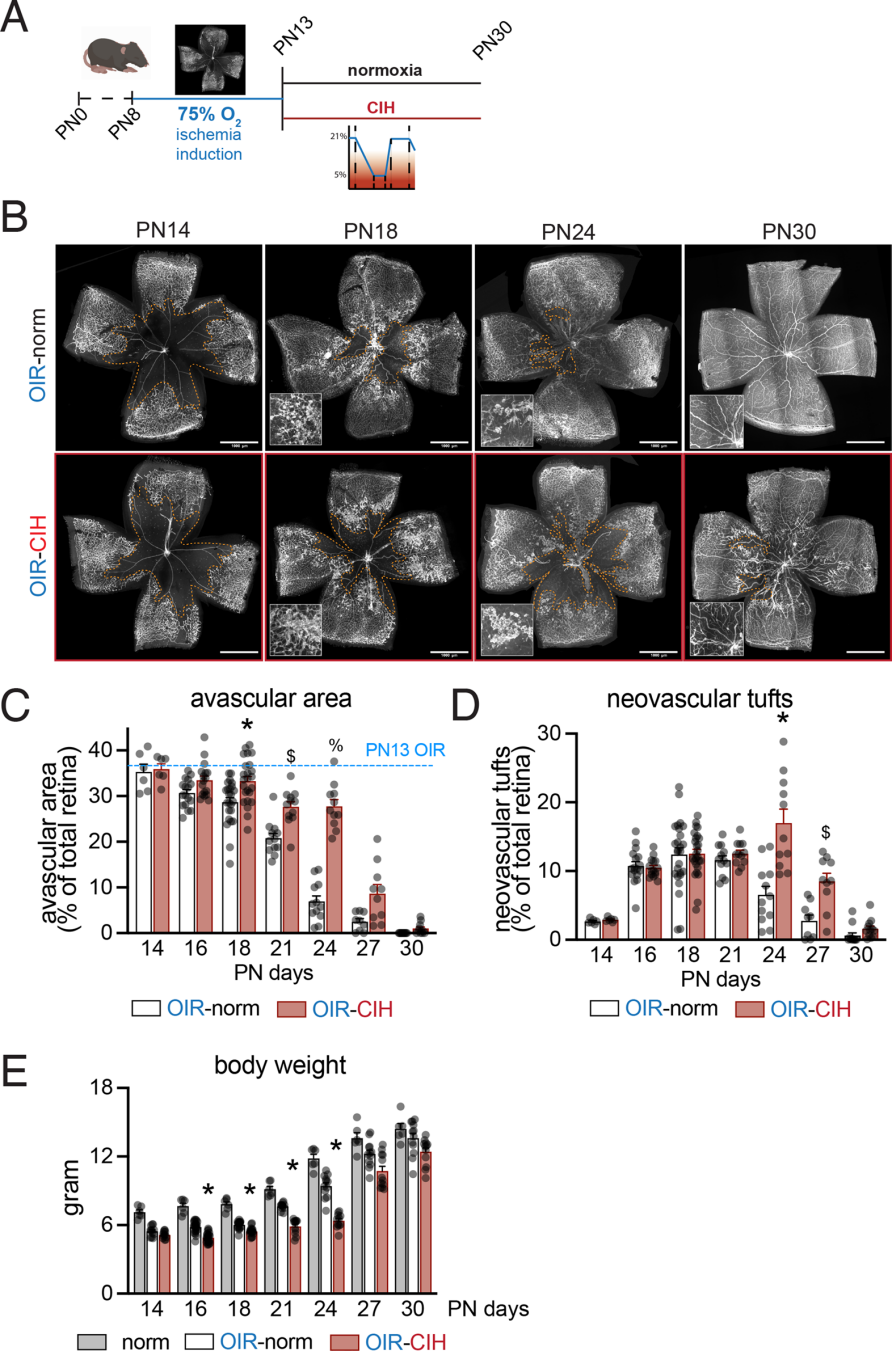
CIH inhibits the revascularization of ischemic retina, promotes pathological intravitreal neovascularization, and induces a transient growth retardation. (**A**) Schematic of the time course of OIR, normoxia, and CIH conditions. (**B**) Representative retinal flatmounts labeled with isolectin to visualize vessels. Avascular areas are indicated with a dashed orange line. PN18 insets show examples of neovascularization. PN30 insets highlight abnormal vasculature in some revascularized areas in OIR-CIH retinas. Quantification of (**C**) avascular and (**D**) neovascular regions in retinal flatmounts (*n* = 14–30 retinas per timepoint per group; two-way ANOVA followed by Šídák’s multiple comparisons test **C** * *p* = 0.0125 PN18 OIR-Norm versus OIR-CIH, $ *p* = 0.0014 PN21 OIR-Norm versus OIR-CIH, % *p* < 0.0001, **D** * *p* = 0.0028 PN24 OIR-Norm versus OIR-CIH, $ *p* = 0.0089 PN27 OIR-Norm versus OIR-CIH). In C the average percentage of avascular area at the end of OIR (PN13) is indicated with a blue dashed line. (**E**) Body weight in grams of pups from PN14-PN30 (*n* = 6–12 animals per group; two-way ANOVA followed by Šídák’s multiple comparisons test * *p* < 0.0001 PN16 OIR-Norm versus OIR-CIH, * *p* = 0.0001 PN18 OIR-Norm versus OIR-CIH, * *p* < 0.0001 PN21 OIR-Norm vs. OIR-CIH, * *p* < 0.0001 PN24 OIR-Norm versus OIR-CIH). Scales bars = 1000 μm; all values displayed as mean ± SEM, individual points indicate values per retina (C-D) or per animal (**E**)

### CIH inhibits revascularization and promotes pathological neovascularization in the ischemic retina

Clinical studies show that obstructive sleep apnea is associated with proliferative DR and DME after adjustment to confounders including age, sex, body mass index, and hypertension [14–16], but the underlying mechanism is unknown. To investigate if CIH exacerbates advanced ischemic proliferative retinopathy we used the mouse model of oxygen induced retinopathy (OIR), which induces microvascular degeneration followed by neovascularization, contrary to diabetic animal models that only develop mild microvascular degeneration. Following the 75% O_2_ exposure from PN8-PN13, which induces a central avascular area that subsequently drives the neovascularization [20], pups were exposed to CIH conditions or normoxia conditions (the same gas flow, noise and sleep fragmentation, but with constant 20.9% O_2_) until sacrifice (Fig. 2A).

In this model neovascular tuft development reaches a maximum five days after return to room air (PN 17–18). In parallel, the avascular area disappears as the ischemic retina revascularizes and the neovascular tufts resorb by PN25 [21]. Isolectin-stained retinal flatmounts revealed a similar sized avascular area at PN14, after 24 h of CIH or normoxia exposure. However, while the avascular area of control mice quickly disappeared over the observation period and neovascular tufts were regressing, CIH significantly inhibited the revascularization of the ischemic retina, associated with delayed and exaggerated neovascularization (Fig. 2B). Quantification of the size of the central avascular area revealed significant differences at PN18, PN21, and PN24, with an over three-fold bigger avascular area in the CIH group at PN24 (Fig. 2C). While neovascular tuft formation reached a maximum at PN18 in control mice, CIH exposed animals reached a maximal pathological neovascular response only at PN24 at which point the surface covered by neovascular tufts was twice as large as in control mice (Fig. 2D). At PN30 retinas from the CIH group were still very recognizable by the irregularity of the intra-retinal revascularization and remaining neovascular, while control retinas had, other than some degree of increased vessel tortuosity, renormalized their retinal vasculature, as previously described [21](Fig. 2B).

Interestingly, the transient growth retardation that is observed in OIR-norm mice compared to mice kept under normoxic conditions was also prolonged significantly in OIR-CIH mice, reaching significance at PN21 and 24. However, despite continued CIH, their growth curve had caught up with the other conditions by PN30 (Fig. 2E).

Taken together, although CIH did not affect normal vascular development (Fig. 1) it profoundly inhibited the revascularization of the ischemic retina. The resulting prolongation of the ischemia was associated with more persistent and exaggerated pathological neovascularization. Interestingly, the CIH also accentuated the transient growth retardation induced by OIR, suggesting a possibly more generalized effect on angiogenesis in the growing animals.

### CIH aggravates photoreceptor segment degeneration and long-lasting retinal dysfunction

Additionally to neovascularization, pathological neuronal degeneration occurs in the inner retina during OIR [35, 36], ROP [4], and DR [5, 6]. Optical coherence tomography (OCT) studies revealed that the photoreceptor segments undergo degenerative changes in DR. The line that represents the junction of the mitochondria-containing inner segments and photo-sensitive outer segments (IS/OS) gets disrupted [7], and photoreceptor outer segments shorten [8], which strongly correlated with visual function in these patients [9].

**Fig. 3:**
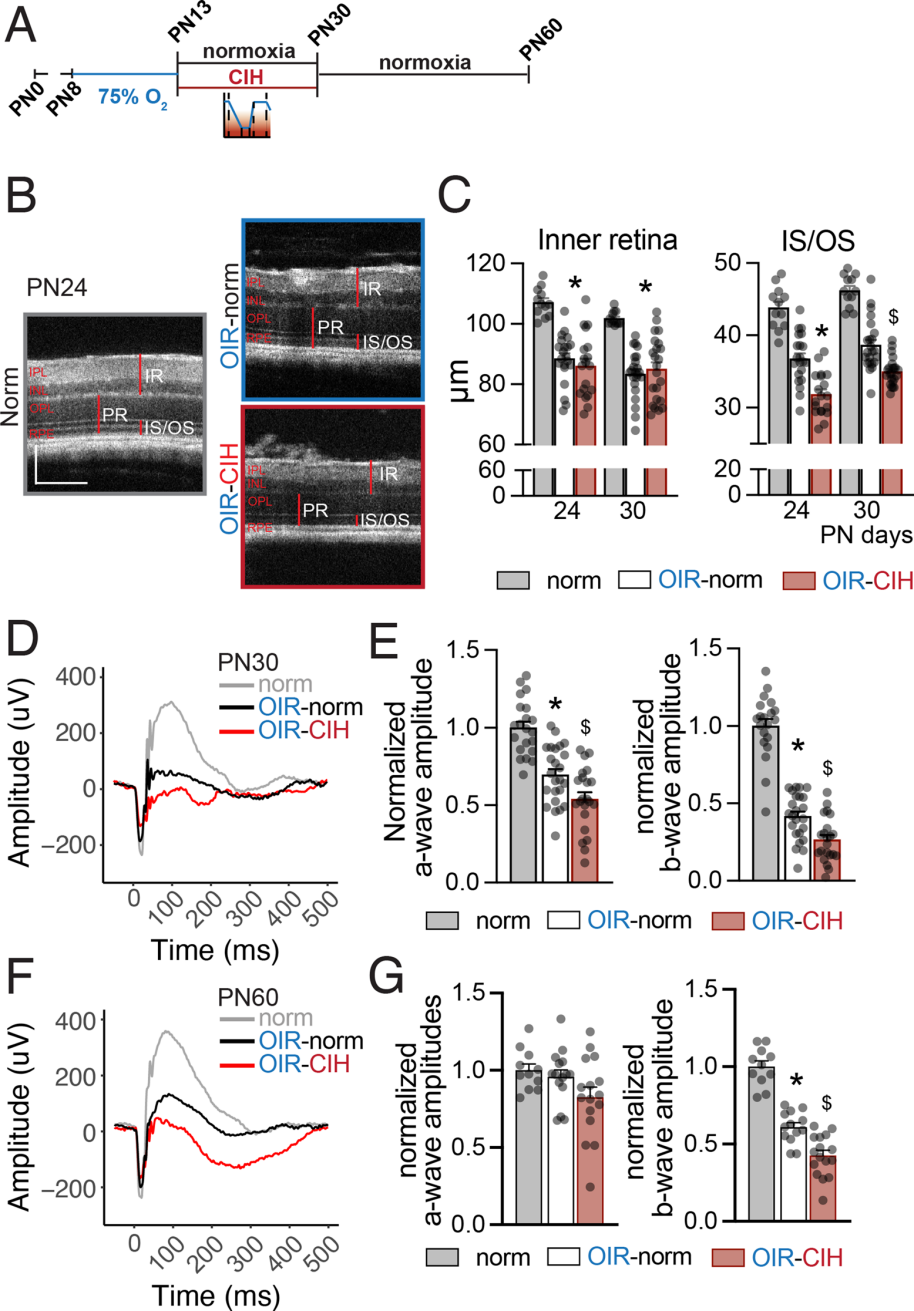
OIR induces neurodegeneration and CIH exacerbates functional deficits in retinal signaling. (**A**) Schematic of the time course of OIR, normoxia, and CIH conditions. (**B**) Representative B scans of Norm, OIR-Norm, and OIR-CIH retinas at PN24. B scans are from the central retina, near the optic nerve head. Horizontal scale bar = 200 μm, vertical scale bar = 150 μm. (**C**) Quantification of the thickness of the inner retina, ONL, and IS/OS layers, measured from the OCT B scans (*n* = 12–24 eyes per group; one-way ANOVA followed by Šídák’s multiple comparisons test, inner retina * *p* < 0.0001 PN24 and PN30 Normoxia vs. OIR-Norm and Normoxia vs. OIR-CIH; ONL * *p* = 0.0034 PN24 Normoxia vs. OIR-CIH; IS/OS * *p* < 0.0001 PN24 Normoxia vs. OIR-Norm, Normoxia vs. OIR-CIH, and OIR-Norm vs. OIR-CIH, * *p* < 0.0001 P30 Normoxia vs. OIR-Norm and Normoxia vs. OIR-CIH, $ *p* = 0.0007 P30 OIR-Norm vs. OIR-CIH). (**D**) Representative ERG waveforms of normoxia (grey), OIR-norm (black), and OIR-CIH (red) at PN30 in response to 4 ms flashes of 3 cd*s/m^2^. (**E**) Quantification of a- (left) and b-wave (right) amplitudes at PN30 in response to 4 ms flashes of 3 cd*s/m^2^ (*n* = 20–24 eyes per group; one-way ANOVA followed by Šídák’s multiple comparisons test, normalized a-wave amplitude * *p* < 0.0001 Normoxia vs. OIR-Norm, $ *p* < 0.0001 Normoxia vs. OIR-CIH and *p* = 0.0205 OIR-Norm vs. OIR-CIH; normalized b-wave amplitude * *p* < 0.0001 Normoxia vs. OIR-Norm, $ *p* < 0.0001 Normoxia vs. OIR-CIH and *p* = 0.0096 OIR-Norm vs. OIR-CIH). (**F**) Representative ERG waveforms of normoxia (grey), OIR-norm (black), and OIR-CIH (red) at PN60 in response to 4 ms flashes of 3 cd*s/m^2^. (**G**) Quantification of a- (left) and b-wave (right) amplitudes at PN60 in response to 4 ms flashes of 3 cd*s/m^2^ (*n* = 11–16 eyes per group; one-way ANOVA followed by Šídák’s multiple comparisons test, normalized a-wave amplitude all p values > 0.05, normalized b-wave amplitude * *p* < 0.0001 Normoxia vs. OIR-Norm, $ *p* < 0.0001 Normoxia vs. OIR-CIH and *p* = 0.0008 OIR-Norm vs. OIR-CIH). All values displayed as mean ± SEM, individual points indicate values per eye. IPL = inner plexiform layer, INL = inner nuclear layer, OPL = outer plexiform layer, ONL = outer nuclear layer, RPE = retinal pigment epithelium, IR = inner retina, PR = photoreceptors, IS/OS = inner and outer segments

In mice, OCT at PN24 (Fig. 3A and B) and PN30 confirmed [35] inner retinal thinning in the ischemic retina compared to the normal retina, but this was not affect by CIH (Fig. 3C, left). However, CIH consistently and significantly worsened the OIR-induced thinning of the photoreceptor segment layer (IS/OS) at PN24 and PN30 (not the outer nuclear layer that contains the photoreceptor nuclei)(Fig. 3C, right).

Functionally, electroretinography (ERG) examination at PN30 revealed that CIH significantly reduced the amplitude of OIR-induced scotopic a-waves, a measure of rod photoreceptor function, and the subsequent b-waves, a measure of transmission to and function of the inner retina (Fig. 3D and E). Importantly, animals were then kept in the normal animal facility from PN30 to PN60, and although the segment length had normalized (data not shown), the CIH-induced decrease in function was still detectable at PN60, 30 days after cessation of CIH (Fig. 3F and G).

In summary, our data demonstrate that CIH induces the degeneration of photoreceptor segments in addition to the previously described OIR-induced thinning of the inner retina. Further, these morphological differences were associated with lasting deficits in retinal signaling, which are exacerbated by CIH even after return to normal oxygen conditions.

### CIH dampens the reactive microglial expansion in ischemic retinopathy

**Fig. 4:**
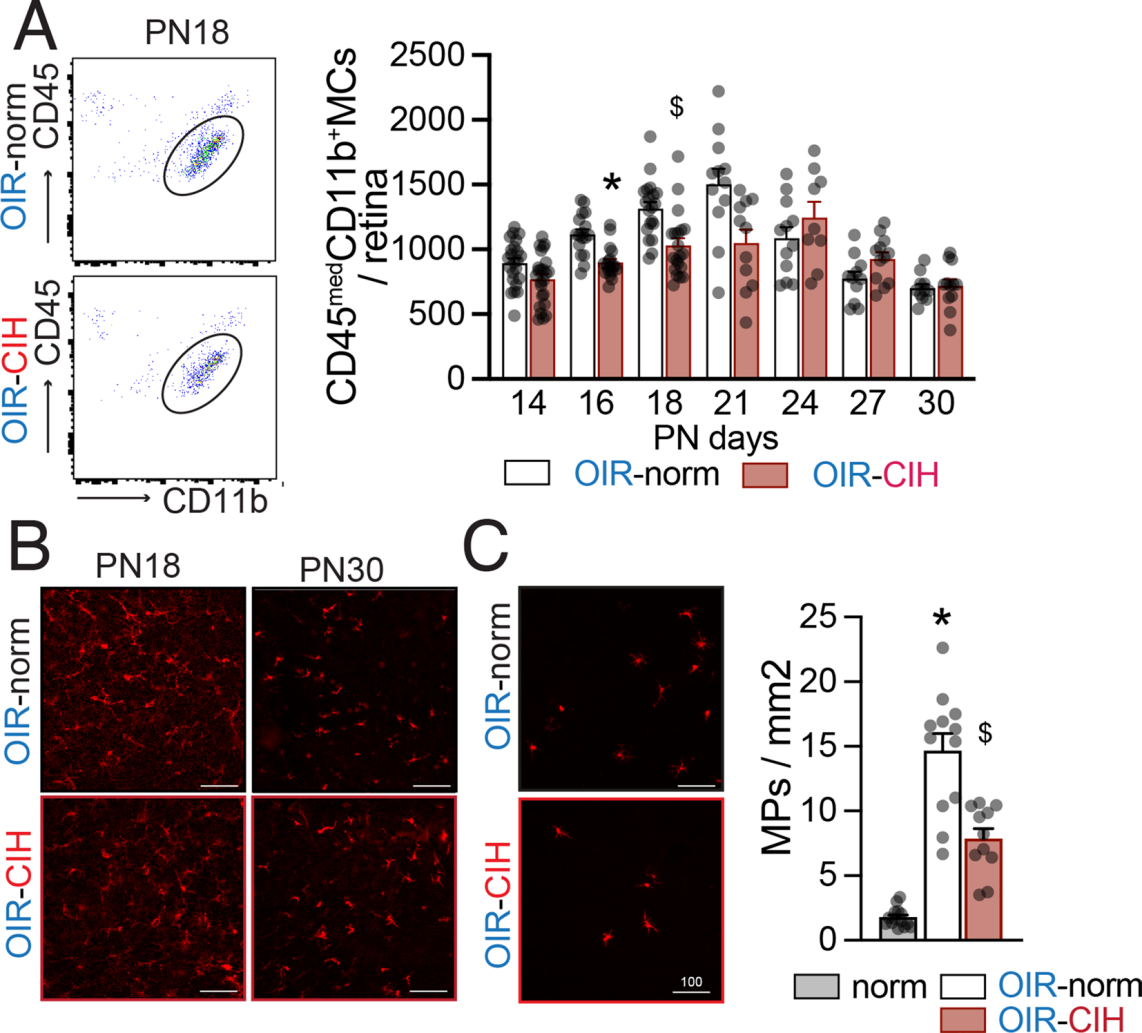
CIH dampens the reactive microglial expansion in ischemic retinopathy. (**A**) Left: Representative gating of CD45^med^CD11b^+^ microglial cells (MC). Right: Quantification of single alive CD45^med^CD11b^+^ retinal microglia (*n* = 11–24 retinas per group; two-way ANOVA and Šídák’s multiple comparisons test * *p* = 0.0006 PN16 OIR-Norm versus OIR-CIH, $ *p* = 0.0039 PN18 OIR-Norm versus OIR-CIH). (**B**) Representative images of central inner retinal microglia, labeled with IBA1 (red) in retinal flatmounts at PN18 and PN30. (**C**) Left: Representative images of subretinal mononuclear phagocytes (MPs), labeled with IBA1 (red) on RPE/choroidal flatmounts at PN30. Right: quantification of subretinal IBA1 + MPs at PN30 (*n* = 11–14 eyes per group; one-way ANOVA followed by Šídák’s multiple comparisons test, * *p* < 0.0001 Normoxia vs. OIR-Norm, $ *p* < 0.0001 Normoxia vs. OIR-CIH and OIR-Norm vs. OIR-CIH). Scales bars = 100 μm; all values displayed as mean ± SEM, individual points indicate values per retina

In rodents, revascularization of the ischemic retina in OIR has been shown to be dependent on microglial cells [25, 26]. Using flow cytometry, we did not detect a significant infiltration of monocytes or other leukocytes (data not shown). Quantification of retinal microglia numbers revealed a quick increase in microglia in the ischemic phase of the OIR model, peaking at PN21, which then returned to baseline by PN27 in the OIR-norm group. In OIR-CIH this reactive swell of the microglia population was significantly suppressed (Fig. 4A). Additionally, microglia visualized in IBA1-labelled retinal flatmounts at PN18 of the central retina revealed that microglia of the ischemic retina of OIR-CIH retinas lacked the transient morphological signs of activation (shortened processes, larger cell bodies) observed at PN18 in OIR-Norm microglia, which normalized by PN30 (Fig. 4B). Similarly, the infiltration of IBA1 + macrophages in the subretinal space that is physiologically devoid of macrophages was significantly blunted in OIR-CIH animals compared to the OIR-Norm group (Fig. 4C). Given the absence of monocyte-derived macrophages in cytometry these cells were likely microglia, similar to the accumulation of protective microglial cells previously described in other photoreceptor disease [37, 38].

In summary, these data demonstrate the CIH inhibits the expansion and activation of retinal microglia in the ischemic retina that has been shown to be associated with the reparative revascularization of the ischemic retina [25] and could have a protective role on photoreceptors [37].

### CIH reduces retinal *Csf1* transcription

**Fig. 5:**
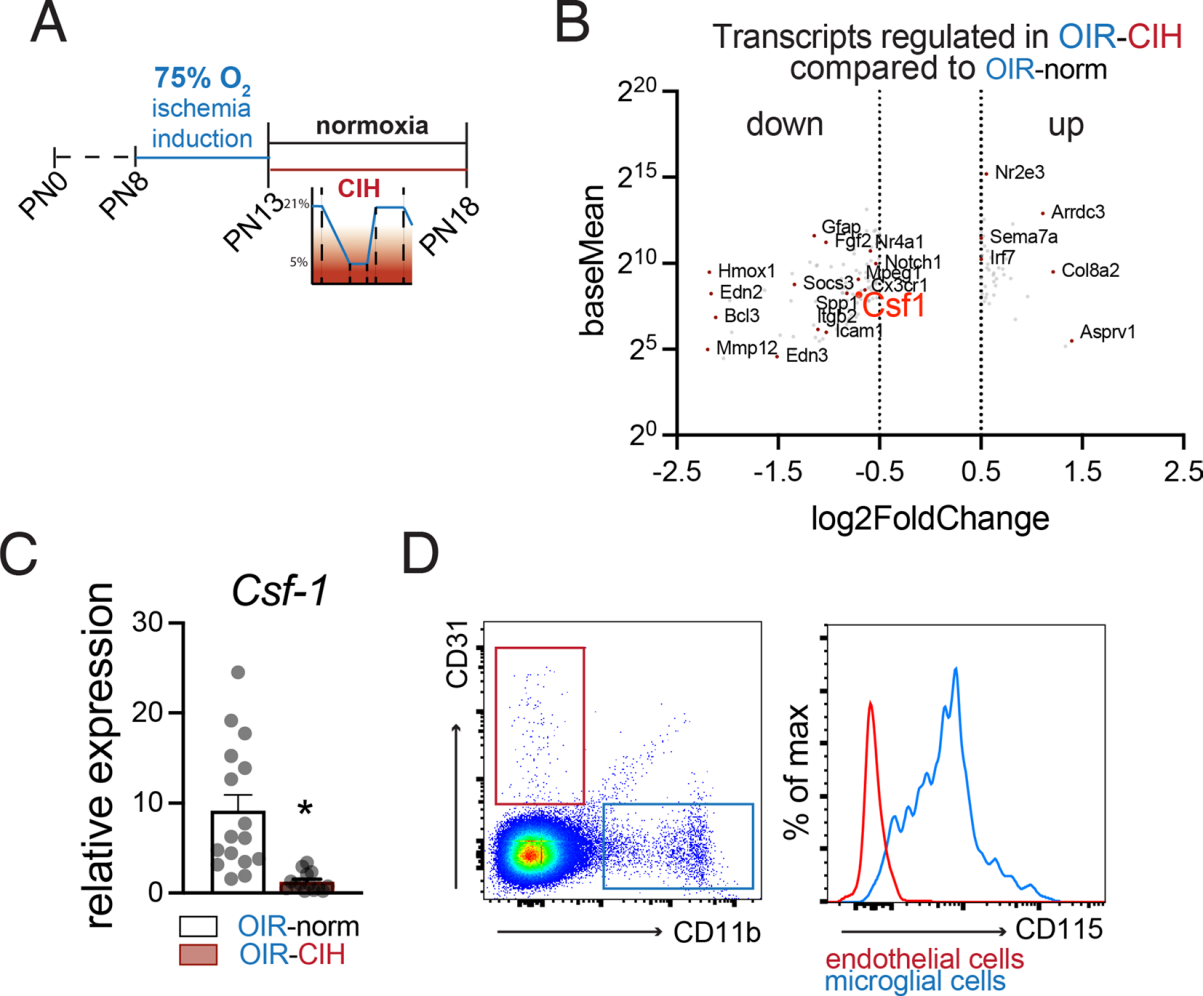
CIH reduces retinal *Csf1* transcription. (**A**) Schematic of the time course of OIR, normoxia, and CIH conditions. (**B**) Volcano plot of differentially expressed genes detected in bulk RNA sequencing of OIR-Norm and OIR-CIH retinas. (**C**) Comparison of *Csf1* expression in OIR-Norm and OIR-CIH retinas at PN18 by qPCR (*n* = 16 retinas per group; Mann-Whitney test * *p* < 0.0001 OIR-Norm versus OIR-CIH; all values displayed as mean ± SEM, individual points indicate values per retina). (**D**) Left: Flow cytometry gating scheme for CD31 + endothelial cells and CD11b + microglia. Right: CD115 (CSF1R) expression on single alive CD31 + endothelial cells (red) and single alive CD45^med^CD11b^+^ microglia (blue)

To identify potential underlying transcriptional differences, we next performed bulk RNA sequencing (RNAseq) on OIR-Norm and OIR-CIH retinas at PN18 (Fig. 5A). The differentially expressed genes (Fig. 5B, Supplemental Table 1) included CIH down-regulated *Hmox1*, *C4b*, *Cd84*, all previously associated with immune functions. Indeed, we performed a Gene Ontology enrichment analysis and found that many of the top enriched GO terms were related to immune responses (Supplemental Fig. 1).

**Fig. 6:**
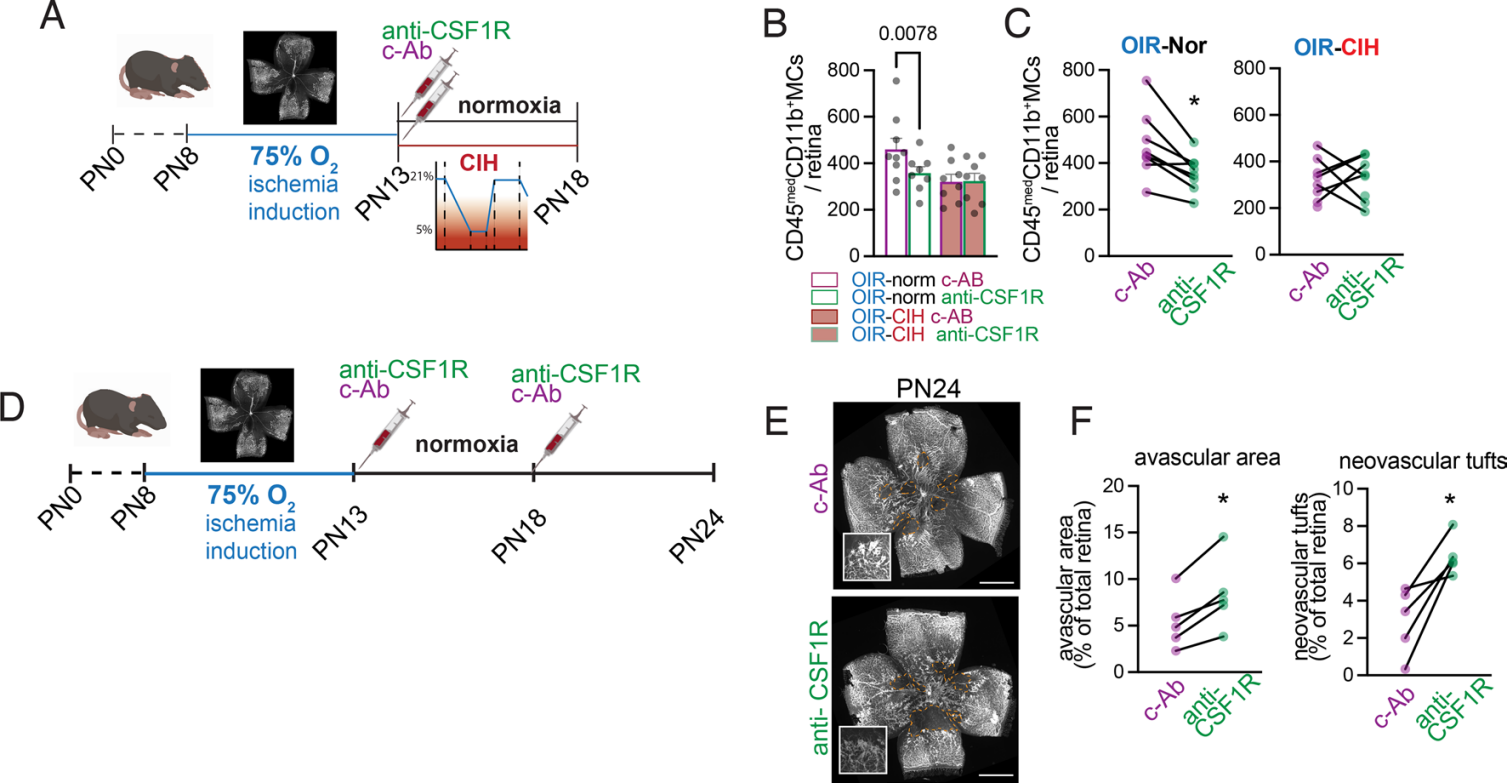
CSF1R inhibition during the ischemic phase of OIR recapitulates the effect of CIH on microglia and vasculature (**A**) Schematic of the time course of injections, and OIR, normoxic, and CIH conditions for the data shown in B-C. (**B**) Quantification of CD45^med^CD11b^+^ microglia by flow cytometry at PN18, 5 days after intravitreal antibody injection, comparing across all experimental groups (*n* = 8 retinas per condition; values displayed as mean ± SEM, individual points indicate values per retina). (**C**) Quantification of CD45^med^CD11b^+^ microglia by flow cytometry, as in B, but showing the paired comparisons between contralateral eyes within the OIR-Norm (left) and OIR-CIH (right) groups (*n* = 8 pups per group; paired Mann-Whitney test * *p* = 0.0078 OIR-Norm c-Ab versus anti-CSF1R). (**D**) Schematic for the time course of injections and OIR and normoxic conditions for the data shown in E-F. (**E**) Representative flatmounts of PN24 retinas after anti-CSF1R antibody or control injections. Vessels are labeled with isolectin and avascular areas are indicated with dashed yellow lines. Insets shown representative areas of neovascularization (NV). (**F**) Quantification of avascular (left) and neovascular tuft (right) area on the retinal flatmounts (*n* = 5 pups per group; paired Mann-Whitney test * *p* = 0.0312 c-Ab versus anti-CSF1R avascular area, * *p* = 0.0312 c-Ab versus anti-CSF1R neovascular tufts). Scale bars = 1000 μm

One of the genes down-regulated in OIR-CIH retinas was colony stimulating factor 1 (*Csf1)*, a very important regulator of macrophage/microglia half-life [39]. RT-QPCRs confirmed this very significant down-regulation in *Csf1* mRNA in OIR-CIH compared to OIR-Norm mice at PN18 (Fig. 5C), which could therefore be responsible for the lack of microgliosis observed in the OIR-CIH group. Cytometric analysis of CD115 (the CSF1 receptor, CSF1R) expression on both retinal microglia and endothelial cells from OIR-Norm PN18 retinas showed that endothelial cell CD115 expression was negligible and that microglia were the main CD115 expressing cells (Fig. 5D), suggesting that any effects of decreased *Csf1* expression are mediated via signaling on retinal microglia.

### CSF1R inhibition during the ischemic phase of OIR recapitulates the effect of CIH on microglia and vasculature

To determine if decreased CSF1-CSF1R signaling could drive several of the effects of CIH on the ischemic retina, we used an anti-CSF1R blocking antibody to inhibit CSF1R. Immediately following the induction of OIR (PN13), pups received an injection of the anti-CSF1R antibody in one eye and an IgG isotype control in the contralateral eye (Fig. 6A). The CSF1R blocking in the OIR-norm group decreased the microglia population to levels equivalent to the size as in OIR-CIH. Injections of the anti-CSF1R antibody in the OIR-CIH group, characterized by an already downregulated *Csf1*, had no effect on the number of retinal microglia (Fig. 6B group comparison, Fig. 6C right-eye versus left-eye paired comparison).

To determine whether decreasing microglia via CSF1-CSF1R signaling in ischemic retinas could recapitulate the exacerbated vascular pathology induced by CIH, we intravitreally injected anti-CSF1R antibody in one eye and isotype control in the contralateral eye immediately following oxygen exposure, and kept the mice in normoxia conditions (Fig. 6D). CSF1R blocking antibody injected eyes developed larger avascular areas and persistent neovascularization compared to their contralateral control antibody injected eyes (Fig. 6E-F), similar to eyes of the OIR-CIH group.

Taken together our data shows that the inhibition of CSF1-CSF1R signaling in OIR-Norm animals recapitulates the vascular phenotype that is observed in OIR-CIH mice in which we showed the *Csf1* expression was decreased.

## Discussion

Using a combination of oxygen-induced microvascular degeneration that induces an ischemic retina and experimental CIH, we here investigated the mechanisms that could link apnea of prematurity with the risk of severe proliferative ROP [11, 12] and obstructive sleep apnea (OSA) with an increased prevalence of severe proliferative DR [14–16].

The OIR mouse model uses continuous oxygen exposure in the vaso-obliterative phase, contrary to the rat model which necessitates alternating hyperoxia–hypoxia cycles, where the oxygen levels cycle between 50 and 10% every 24 h for the first 14 d after birth (for review see Liu et al. [28]. The mouse model thereby more clearly separates the hyperoxic, vaso-obliterative phase from the ischemic phase, contrary to the rat model in which an aspect of intermittent hypoxia is already introduced from the beginning. The OIR mouse model also very reproducibly develops neovascularizations of the ischemic retina that characterize severe human ROP and DR, contrary to rodent animal models of diabetes that only model early stages of non-proliferative retinopathy [27].

Apnea of prematurity and OSA produce repeated episodes of hypoxemia followed by re-oxygenation, called chronic intermittent hypoxia (CIH), believed to be the major contributor to apnea-related morbidity [19]. We experimentally replicated CIH using 10 h daily of 266 cycles of quick oscillations of the O_2_ concentration in the cage during their sleep [29], which corresponds to severe obstructive sleep apnea. Remarkably, this CIH regimen on its own did not induce any phenotype in normal mice (Fig. 1), similar to human patients in whom even severe OSA only causes mild vascular changes in the absence of other underlying retinal diseases [36].

In contrast, CIH profoundly inhibited the previously described revascularization of the ischemic retina [21], resulting in an important, over three-fold larger avascular area at PN24 (Fig. 2). This vascular growth retardation was mirrored by a general growth retardation in the CIH exposed OIR pups, a phenomenon we did not observe in pups without prior hyperoxia exposure (Fig. 1). This growth retardation might be the result of altered food intake, but could also suggest that CIH can more generally inhibit angiogenesis after the vasculature has been rendered vulnerable by the hyperoxic phase. In the retina, the resulting prolongation of the ischemia by CIH was likely responsible for the more persistent and exaggerated pathological neovascularization observed at PN24 and PN27 (Fig. 2), a previously described mechanism [40]. While the vascular changes in the OIR-norm group were largely reversible, as previously described [21] and much like vascular changes in non-severe ROP, the vasculature of the CIH group remained still very recognizably altered due to the irregularity of the intra-retinal revascularization and remaining neovascular tufts at PN30.

Importantly, CIH also aggravated the OIR-induced thinning of the layer of the photoreceptor segments (Fig. 3) in addition to the thinning of the inner retina previously known to be associated with the avascular area in the OIR model [35, 36]. Interestingly, photoreceptor segment changes have also been described in severe DR and DME [7–9], which are associated with sleep apnea [14–16]. Although the human fovea with its foveal cones is anatomically very different from the mouse retina, the perifoveal macula is quite similar to the mouse retina, and it is tempting to speculate that photoreceptor segment changes in severe DR might also be linked to CIH. In our model, these observed photoreceptor segment morphological changes were likely responsible for the CIH-induced further reduction in the scotopic a-wave amplitude and likely contributed to the b-wave amplitude reduction that we confirm OIR induces in mice [10]. Interestingly, the CIH-induced decrease in scotopic responses remained detectable even after 30 days of recovery in room air.

In peripheral disease such as atherosclerosis, CIH is often associated with inflammation and macrophage activation [41]. However, our bulk RNA sequencing of CIH-exposed mice and their controls revealed a downregulation of a number of inflammation-associated genes in the CIH-exposed retina. Interestingly, our analysis revealed a strong downregulation of the macrophage colony-stimulating factor *Csf1*, a cytokine that regulated microglial cell populations [39], which we confirmed by RT-PCR (7-fold decrease; Fig. 5). Our analysis revealed that CSF1R was mainly expressed by microglia while its expression on vascular endothelial cells was negligible, making a direct effect of the lack of CSF-1 on endothelial cells unlikely (Fig. 5). Accordingly, we found the decreased CSF-1 expression in the CIH group blunted the reactive microgliosis and reduced the subretinal microglial cell infiltration observed under normoxic conditions (Fig. 4). These lower microglial cell counts are in accordance with a previous study that showed that CIH reduced microglia proliferation in vitro [42].

In human proliferative DR, intra-ocular CSF-1 concentration are increased compared to non-diabetic patients [43, 44] and Zeng et al. showed a marked increase of inner retina mononuclear phagocytes using general MP markers (HLA-DR, CD45 and CD68) [45], which is likely due to a reactive microgliosis additionally to monocyte derived macrophages that have been shown to drive the disease [46]. Further studies are needed to determine whether sleep apnea influences these CSF1 levels and the associated microgliosis and how these parameters compare between proliferative and non-proliferative DR.

Others and we have previously shown that the revascularization of ischemic retina in OIR is dependent on microglial cells in vivo [25, 26] and human microglial cells enhance migration and tube formation of human retinal endothelial cells in a CSF-1-dependent manner in vitro [44]. To test whether a reduction of CSF1-CSF1R signaling could be responsible for the inhibition of retinal revascularization via a decreased microglial cell population, we treated OIR mice during the ischemic phase with intravitreal CSF1R-blocking antibody in one eye and an isotypic control antibody in the other eye. Compared to tonic, systemic CSF1R inhibition, which is well-known to fully eliminate microglia if administered continuously over a period of around 7 to 14 days, we here chose to inhibit CSF1R signaling by one intravitreal injection of a CSF1R-blocking antibody, which does not cause full microglia elimination but dampens microgliosis and microglial responses as shown in a model of retinal ischemia/reperfusion injury [47]. We chose this approach to be able to evaluate the local rather than systemic CSF1R inhibition and to compare CSF1R inhibition in one eye to the untreated fellow eye in the same animal. In OIR-Norm animals blocking CSF1R induced a decrease in the microglial population to a similar level observed in OIR-CIH mice. Blocking CSF1R in OIR-CIH mice had no effect, in accordance with the observed concurrent down-regulation of *Csf1* in this group which likely already decreased CSF1-CSF1R signaling. Interestingly, the CSF1R-blocking antibody also inhibited the revascularization of the ischemic retina and aggravated pathological neovascularization compared to the control injected contralateral eyes in the OIR-Norm group. The observation that local blocking of CSF1-CSF1R signaling alone is sufficient to recapitulate many of the effects of CIH on the ischemic retina highlights the importance of this local deregulation. Indeed, these experiments suggest that the inhibition of retinal revascularization is not the consequence of the observed systemic growth retardation and also independent of hypertension or eventual pro-inflammatory changes in the periphery. Similarly, the association of obstructive sleep apnea with proliferative DR and DME in humans was shown to be independent of confounders including body mass index and hypertension [14–16].

Interestingly, a CSF-1-dependent microgliosis has also been demonstrated in a streptozotocin-induced model of non-proliferative diabetic retinopathy, which does not develop intra-retinal revascularization or neovascularization [44]. In the OIR model, a previous study that used osteopetrotic mice (op/op mice carrying a mutation of the *Csf1* gene) and a c­fms tyrosine kinase inhibitor reported that CSF-1 inhibition strongly inhibited vitreal neovascularization, but not retinal revascularization [48]. These differences might be explained by the fact that their study analyzed retinae at an earlier time point, PN16, only and it is not clear to what degree op/op mice that lack CSF-1 might congenitally compensate for this decreased expression by the expression of the alternative CSF1R ligand IL34.

## Conclusions

In our study, we uncovered a novel mechanism by which chronic intermittent hypoxia (CIH), which is believed to be the major contributor to sleep apnea-related morbidity, exerts its detrimental effect on neurodegeneration and pathological vascular alterations in ischemic proliferative retinopathy. Our findings unveil that CIH disrupts a protective reactive microgliosis via the downregulation of colony stimulating factor 1 (CSF-1) in ischemic proliferative retinopathy. Thereby CIH reduces intraretinal vasoprotection, increases pathological neovascularization, and exacerbates photoreceptor degeneration and retinal dysfunction. Our findings help to explain the previously unexplained association of sleep apnea with the increased prevalence of severe DR [14–16] and ROP [11, 12]. These results may also have implication for other neurodegenerative diseases linked to sleep apnea, such as Alzheimer’s and Parkinson’s diseases [49, 50], which involved a protective microgliosis [51–53]. Together, our study opens new therapeutic avenues and most importantly underscores the importance of detecting and treating apnea in DR and ROP to help prevent sight threatening severe disease.

## Electronic supplementary material

Below is the link to the electronic supplementary material.

Supplementary Material 1

Supplementary Material 2

## Data Availability

The datasets used and analyzed during the current study are available from the corresponding author on reasonable request. The RNAseq dataset generated during the current study is available in the GEO repository, accession number GSE268420.

## Abbreviations

Ab: Antibody
CIH: Chronic intermittent hypoxia
DME: Diabetic macular edema
DR: Diabetic retinopathy
ERG: Electroretinogram
INL: Inner nuclear layer
IPL: Inner plexiform layer
IR: Inner retina
IS/OS: Inner–and outer–segment
MP: Mononuclear phagocyte
Norm: Normoxia
OCT: Optical coherence tomography
OIR: Oxygen–induced retinopathy
OPL: Outer plexiform layer
OSA: Obstructive sleep apnea
PN: Postnatal
PR: Photoreceptors
RNAseq: RNA sequencing
ROP: Retinopathy of prematurity
RPE: Retinal pigment epithelium
