# Supplementary material for: Chronic intermittent hypoxia disrupts protective microgliosis in ischemic proliferative retinopathy
Source: J Neuroinflammation. 2025 Mar 14;22:82. doi: 10.1186/s12974-025-03392-9 (PMC11909870; doi:10.1186/s12974-025-03392-9)
Supplement: Supplementary file 1 — Supplementary Material 1 [file 12974_2025_3392_MOESM1_ESM.docx]

### **Supplemental Table 1:** Significantly differentially expressed genes between OIR-Norm and OIR-CIH retinas.

List of genes significantly down-regulated (top) or up-regulated (bottom) in OIR-CIH retinas compared to OIR-Norm retinas. Genes are ordered from largest to smallest fold change.

| Gene | log2(Fold Change) | P value |
| --- | --- | --- |
| *Mmp12* | -2.19546 | 2.47E-06 |
| *Hmox1* | -2.17937 | 6.10E-76 |
| *Edn2* | -2.16303 | 2.63E-35 |
| *Bcl3* | -2.11643 | 1.89E-21 |
| *Steap4* | -2.03947 | 2.28E-05 |
| *Flnc* | -1.97922 | 0.000574 |
| *Scube1* | -1.95972 | 2.40E-11 |
| *Lad1* | -1.63299 | 2.77E-32 |
| *Edn3* | -1.51173 | 0.000448 |
| *Timp1* | -1.48226 | 1.58E-06 |
| *Tnnt2* | -1.37407 | 1.62E-12 |
| *Serpina3n* | -1.36266 | 1.54E-26 |
| *Cd84* | -1.34968 | 4.4E-06 |
| *Gm33148* | -1.34835 | 0.000536 |
| *Socs3* | -1.33982 | 0.0001 |
| *Cebpd* | -1.18778 | 1.58E-22 |
| *Myh11* | -1.15113 | 0.000644 |
| *Gfap* | -1.14415 | 2.13E-26 |
| *Gna14* | -1.13474 | 0.000107 |
| *Tgm2* | -1.13009 | 6.42E-10 |
| *Pcolce* | -1.12208 | 3.49E-22 |
| *C4b* | -1.11598 | 5.88E-29 |
| *Itgb2* | -1.10869 | 4.84E-05 |
| *Arhgap30* | -1.09396 | 0.000378 |
| *Gm48237* | -1.06098 | 0.00072 |
| *Fgf2* | -1.02972 | 1.08E-23 |
| *Icam1* | -1.02534 | 0.000508 |
| *Jak3* | -0.99286 | 1.19E-07 |
| *Ptgfr* | -0.99034 | 7.27E-06 |
| *Gm15983* | -0.97834 | 1.47E-15 |
| *Ecel1* | -0.92402 | 0.000201 |
| *Spry3* | -0.91416 | 4.56E-12 |
| *Antxr2* | -0.87876 | 1.01E-15 |
| *Lyz2* | -0.86716 | 4.6E-07 |
| *H19* | -0.86071 | 6.6E-07 |
| *Slc7a11* | -0.85623 | 2.61E-08 |
| *Gm19667* | -0.85504 | 1.79E-07 |
| *Gm20045* | -0.84859 | 0.000404 |
| *Serping1* | -0.83281 | 1.18E-08 |
| *Best3* | -0.82468 | 0.000811 |
| *Spp1* | -0.82154 | 2.81E-08 |
| *Fhad1* | -0.8206 | 2.61E-07 |
| *Mob3c* | -0.8205 | 2.71E-09 |
| *Kif14* | -0.81223 | 0.000397 |
| *B230303O12Rik* | -0.7996 | 0.000182 |
| *Tcf7* | -0.79754 | 0.000512 |
| *2810032G03Rik* | -0.78735 | 0.000392 |
| *Tnfrsf1b* | -0.78105 | 0.000245 |
| *F5* | -0.7781 | 5.57E-05 |
| *Ahnak* | -0.77099 | 2.53E-09 |
| *Ddr2* | -0.76354 | 0.000123 |
| *Sned1* | -0.75921 | 5.55E-10 |
| *Lrrc2* | -0.752 | 5.07E-09 |
| *Slc25a37* | -0.75087 | 3.33E-13 |
| *Gm44037* | -0.73337 | 0.000654 |
| *Vcan* | -0.72789 | 3.62E-06 |
| *Cldn2* | -0.71944 | 0.000248 |
| *Sh2d1a* | -0.71238 | 0.000407 |
| *Mpeg1* | -0.71024 | 9.33E-09 |
| *Gm15513* | -0.70878 | 0.000129 |
| *Csf1* | -0.70338 | 7.35E-05 |
| *Osmr* | -0.69593 | 3.65E-05 |
| *Fcrls* | -0.68488 | 4.22E-06 |
| *Scn7a* | -0.68074 | 3.37E-06 |
| *Cx3cr1* | -0.64342 | 7.12E-05 |
| *Gm20342* | -0.64274 | 0.000206 |
| *Tnfrsf1a* | -0.63731 | 2.65E-05 |
| *Lrrfip1* | -0.63443 | 9.22E-05 |
| *Cp* | -0.62857 | 3.82E-14 |
| *Stat3* | -0.62785 | 1.86E-11 |
| *Fbln2* | -0.62562 | 0.000321 |
| *E2f6* | -0.62409 | 2.24E-09 |
| *Piezo1* | -0.61941 | 0.000558 |
| *Ogn* | -0.61508 | 0.000244 |
| *Arid5a* | -0.61281 | 0.000362 |
| *Col4a6* | -0.60951 | 0.000308 |
| *Marveld3* | -0.6045 | 0.000247 |
| *Fgf2os* | -0.60432 | 0.000327 |
| *Arpc1b* | -0.60113 | 1.06E-05 |
| *Tnip2* | -0.59794 | 3.27E-07 |
| *Nr4a1* | -0.59197 | 1.59E-07 |
| *Sbno2* | -0.58464 | 0.000018 |
| *Nupr1* | -0.58277 | 0.000679 |
| *A2m* | -0.5815 | 1.49E-05 |
| *Col1a2* | -0.57627 | 0.000568 |
| *Matn2* | -0.56825 | 0.000508 |
| *Atp1a2* | -0.55361 | 7.23E-05 |
| *Loxl4* | -0.55298 | 6.98E-06 |
| *Myo10* | -0.54106 | 1.48E-09 |
| *Pde3b* | -0.54083 | 2.68E-05 |
| *Pprc1* | -0.53903 | 0.000011 |
| *Notch1* | -0.53741 | 2.78E-06 |
| *Pros1* | -0.52998 | 0.000051 |
| *Eif4e3* | -0.52939 | 0.000413 |
| *Klhl29* | -0.52778 | 1.9E-07 |
| *Greb1* | -0.52628 | 0.00016 |
| *Bcl6* | -0.5102 | 3.45E-05 |
| *Lama5* | -0.50698 | 0.000229 |
| *Bub1b* | -0.50344 | 1.79E-05 |
| *Rfx2* | -0.50239 | 1.35E-05 |
| *Jag1* | -0.50006 | 8.09E-10 |

| Gene | log2(Fold Change) | P value |
| --- | --- | --- |
| *Asprv1* | 1.396966 | 0.000405 |
| *Upk3b* | 1.334502 | 0.000197 |
| *Col8a2* | 1.213317 | 7.31E-15 |
| *Arrdc3* | 1.110585 | 3.83E-33 |
| *Hmgb2* | 0.971857 | 2.04E-13 |
| *Gm26670* | 0.961857 | 5.12E-11 |
| *Tfap2e* | 0.838723 | 8.83E-05 |
| *Hist1h1c* | 0.812708 | 9.37E-12 |
| *Ciart* | 0.79332 | 2.28E-09 |
| *Bhlhe23* | 0.749628 | 4.15E-11 |
| *Id1* | 0.726885 | 1.6E-08 |
| *Wfikkn2* | 0.716414 | 6.23E-06 |
| *Ankrd37* | 0.674222 | 6.94E-11 |
| *Neurog2* | 0.652274 | 2.65E-07 |
| *Oasl2* | 0.643976 | 2.66E-07 |
| *Tifa* | 0.628751 | 6.58E-06 |
| *Insm1* | 0.628095 | 1.30E-09 |
| *Slc16a8* | 0.62809 | 0.000473 |
| *Id2* | 0.62746 | 6.38E-07 |
| *Gm27032* | 0.62144 | 5.32E-06 |
| *Ppp1r3c* | 0.597896 | 6.84E-10 |
| *Bsnd* | 0.582821 | 0.000164 |
| *Prdm13* | 0.58005 | 1.81E-06 |
| *Pim3* | 0.569174 | 2.27E-09 |
| *Selenbp1* | 0.565297 | 1.32E-08 |
| *2410006H16Rik* | 0.560945 | 0.000261 |
| *2810410L24Rik* | 0.55609 | 0.00034 |
| *Nr2e3* | 0.552607 | 3.25E-08 |
| *Pcsk9* | 0.549653 | 0.000814 |
| *Gm16551* | 0.543908 | 2.29E-05 |
| *Bhlhe40* | 0.537619 | 1.03E-06 |
| *Gm16958* | 0.53288 | 0.000781 |
| *Tfrc* | 0.53074 | 1.94E-09 |
| *Ubtd1* | 0.524596 | 1.46E-05 |
| *Irf2bp2* | 0.512755 | 6.26E-07 |
| *2310039H08Rik* | 0.510484 | 0.000493 |
| *Sema7a* | 0.503031 | 1.63E-07 |
| *Irf7* | 0.5022 | 3.14E-05 |
| *Usp2* | 0.501731 | 2.86E-08 |
| *Nnat* | 0.501629 | 1.17E-08 |
| *Racgap1* | 0.501533 | 7.65E-05 |
