# Supplementary material for: Chronic intermittent hypoxia disrupts protective microgliosis in ischemic proliferative retinopathy
Source: J Neuroinflammation. 2025 Mar 14;22:82. doi: 10.1186/s12974-025-03392-9 (PMC11909870; doi:10.1186/s12974-025-03392-9)
Supplement: Supplementary file 2 — Supplementary Material 2 [file 12974_2025_3392_MOESM2_ESM.docx]

### **
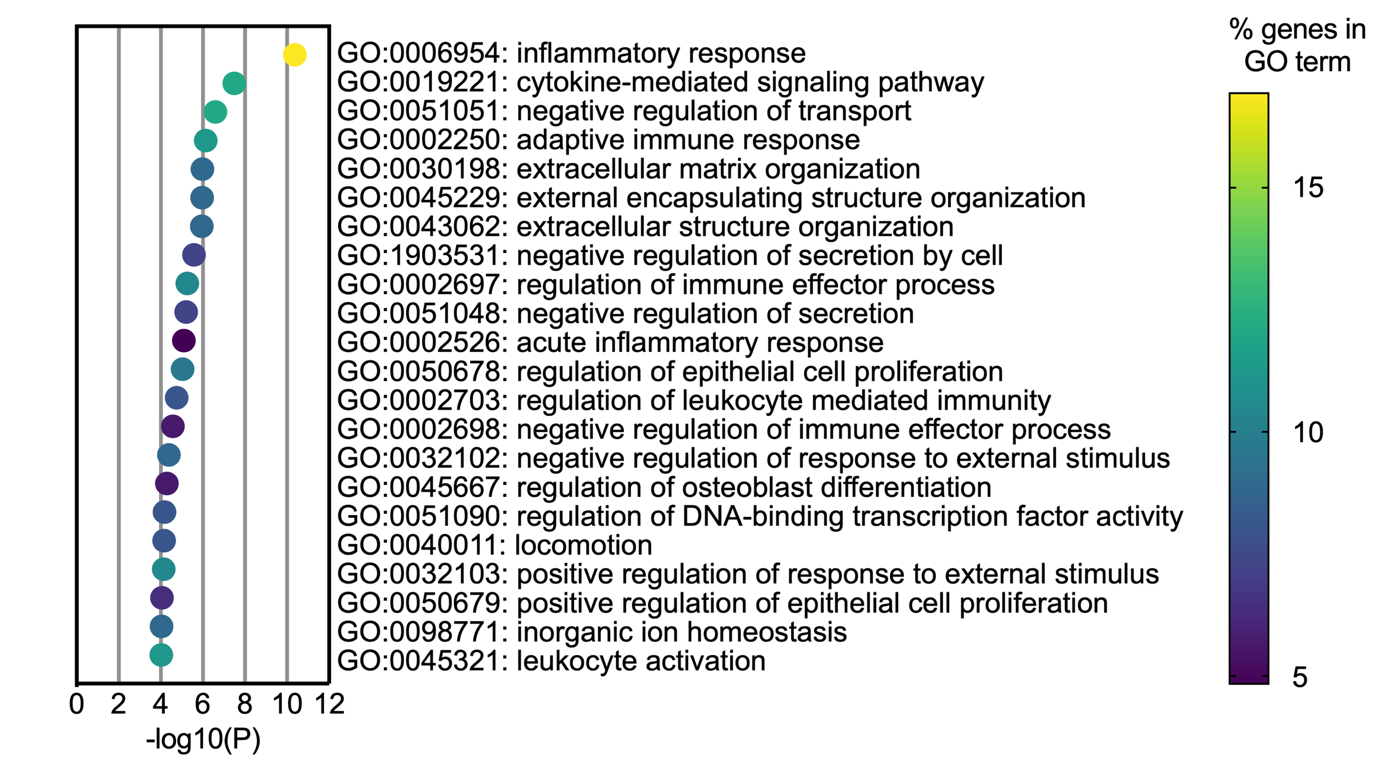
**

### **Supplemental Figure 1:** Top Gene Ontology terms.

List of top Gene Ontology (GO) terms enriched in the list of genes differentially expressed between OIR-Norm and OIR-CIH retinas. Terms are ordered by -log10(P) value and points are color coded by the percentage of genes in the list associated with the corresponding GO term.
